# Discovering myeloid cell heterogeneity in the lung by means of next generation sequencing

**DOI:** 10.1186/s40779-019-0222-9

**Published:** 2019-10-25

**Authors:** Jing-jing Ji, Jie Fan

**Affiliations:** 1 Department of Surgery, University of Pittsburgh School of Medicine, Pittsburgh, PA 15213 USA; 2 Department of Pathophysiology, Southern Medical University, Guangzhou, 510515 China; 3 Research and Development, Veterans Affairs Pittsburgh Healthcare System, Pittsburgh, PA 15240 USA; 4 McGowan Institute for Regenerative Medicine, University of Pittsburgh, Pittsburgh, PA 15219 USA

**Keywords:** Acute lung injury, Lung cancer, Lung disease, Lung development, Single-cell RNA sequencing

## Abstract

The lung plays a vital role in maintaining homeostasis, as it is responsible for the exchange of oxygen and carbon dioxide. Pulmonary homeostasis is maintained by a network of tissue-resident cells, including epithelial cells, endothelial cells and leukocytes. Myeloid cells of the innate immune system and epithelial cells form a critical barrier in the lung. Recently developed unbiased next generation sequencing (NGS) has revealed cell heterogeneity in the lung with respect to physiology and pathology and has reshaped our knowledge. New phenotypes and distinct gene signatures have been identified, and these new findings enhance the diagnosis and treatment of lung diseases. Here, we present a review of the new NGS findings on myeloid cells in lung development, homeostasis, and lung diseases, including acute lung injury (ALI), lung fibrosis, chronic obstructive pulmonary disease (COPD), and lung cancer.

## Background

The lung is constantly exposed to the external environment due to its gas exchange function. This exposure increases the risk of injury from hazardous stimuli in ambient air, such as pathogenic microbes, noxious pollutants, and aspirated gastric contents [1]. Compared with peripheral vasculature, lung vasculature is highly branched, allowing for highly effective gas exchange [2]. In addition, this highly branched vasculature increases the possibility of retention of circulating cells, such as neutrophils and circulating tumor cells (CTCs), making lung susceptible to systemic infectious or sterile stimulus [3, 4]. Therefore, lung inflammation and injury are served as major components of multiorgan dysfunction syndrome in systemic inflammatory responses. Furthermore, the lung is also the most common site of metastatic cancer lesions.

Pulmonary homeostasis is maintained by a network of tissue-resident cells, including epithelial cells, endothelial cells, and myeloid leukocytes. Myeloid cells of the innate immune system form a critical barrier with epithelial cells in the lung. Under normal conditions, resident alveolar macrophages play crucial roles in maintaining the homeostasis of the lung by disposing of inhaled microbes and particulates and by suppressing the development of inappropriate inflammatory and immune responses [5]. Under an inflammatory state, neutrophils quickly respond to the cues secreted from resident macrophages and epithelial cells and are recruited to the inflammatory site. Neutrophils kill invading pathogens in phagosomes by liberating cytotoxic proteins, peptides, and enzymes and activating reactive oxygen species (ROS). Neutrophils are conventionally regarded as terminally differentiated cells with little transcriptional plasticity, as all of their “weapons” are stored in the cells after maturation [6]. If they do not encounter hazardous stimuli, neutrophils undergo apoptosis or are cleared by macrophages, preventing release of their “weapons”. However, with the development of next generation sequencing (NGS), including RNA sequencing and high-content single-cell technologies, a variety of neutrophil subtypes with high transcriptional plasticity have been described [7]. These unbiased analyses are rapidly changing traditional understandings and showing the heterogeneity of myeloid cells in all systems.

Here, we present a review on the new NGS findings of myeloid cells, particularly from single-cell RNA sequencing (scRNA-seq), in lung development, homeostasis, and lung diseases, including acute lung injury (ALI), lung fibrosis, chronic obstructive pulmonary disease (COPD), and lung cancer.

### NGS, scRNA-seq, and myeloid cell profiling

In 1977, Sanger and colleagues [8] published a methodological paper on determining the DNA sequence, providing a tool for deciphering complete genes. This sequencing method, also known as Sanger sequencing, is based on specific chain-terminating inhibitors of DNA polymerase [8, 9] and was subsequently the most widely used sequencing method for the next 30 years. Sanger sequencing was able to obtain the sequence of DNA as long as 1000 bp with high accuracy. However, this low-throughput sequencing method was expensive, time consuming and insufficient to achieve the ultimate goal of deciphering the complicated biological genome. These weaknesses drove the development of high-throughput sequencing: NGS.

NGS can perform millions or even billions of reactions simultaneously, increasing the efficiency of the process. This approach has significantly expanded our knowledge about gene heterogeneity, not only with regard to sequences of DNA and RNA but also their modifications, such as methylation [10]. Different NGS platforms are distinguished by the technologies used in sequencing, including pyrosequencing, sequencing-by-synthesis technology, and ion semiconductor sequencing [11]. The principles and advantages of each platform have been reviewed by Anderson et al. [12] in detail. Common processes shared by these platforms are DNA library construction, sequencing in the machine, and output data analysis. Bioinformatics analysis is used to piece the fragments together by mapping the individual reads to the reference genome. NGS directly shows the gene variants in physiological and pathological states. Combined with newly developed computational tools and published databases, such as String and the Kyoto Encyclopedia of Genes and Genomes (KEGG), further conclusions can be drawn from differentially expressed genes, including prediction of protein-protein interaction and construction of signaling pathways [13]. In turn, the sequencing data also enrich the databases, providing guidance for genetic diseases and clinical diagnostics [14].

Single-cell sequencing (SCS), developed in the last decade, has emerged as a powerful new set of technologies in NGS, including single-cell DNA sequencing, scRNA-seq, and single-cell epigenomic sequencing [15]. Traditional sequencing technologies analyze bulk tissue samples composed of millions of cells. However, most organisms are composed of various cells. These average expression data have difficultly resolving cell-to-cell variations and fail to identify rare cells that may play a crucial role in physiological or pathological progression. SCS methods provide a way to comprehensively profile genetic, epigenetic, spatial, and lineage information in individual cells. Integrating SCS data can construct holistic representations of the cell state, reveal intrinsic regulatory networks, uncover cell-cell crosstalk, identify rare cell types, and predict potential roles of these cells [16]. With the help of SCS, especially scRNA-seq, and bioinformatics analysis, myeloid cell heterogeneity and ontogeny in bone marrow have been revealed, which has been reviewed by Schultze et al. [17] in detail. These new findings have encouraged people to rethink the immune microenvironment in the lung, which consists of resident leukocytes, local stromal cells, and their interactions. Even though published reports are not abundant, several scRNA-seq studies in lung tissue have identified distinct myeloid cell types and their dynamic changes during development and pathological processes [18, 19]. By ligand-receptor mapping, new cell-cell crosstalk has also been revealed.

**Fig. 1:**
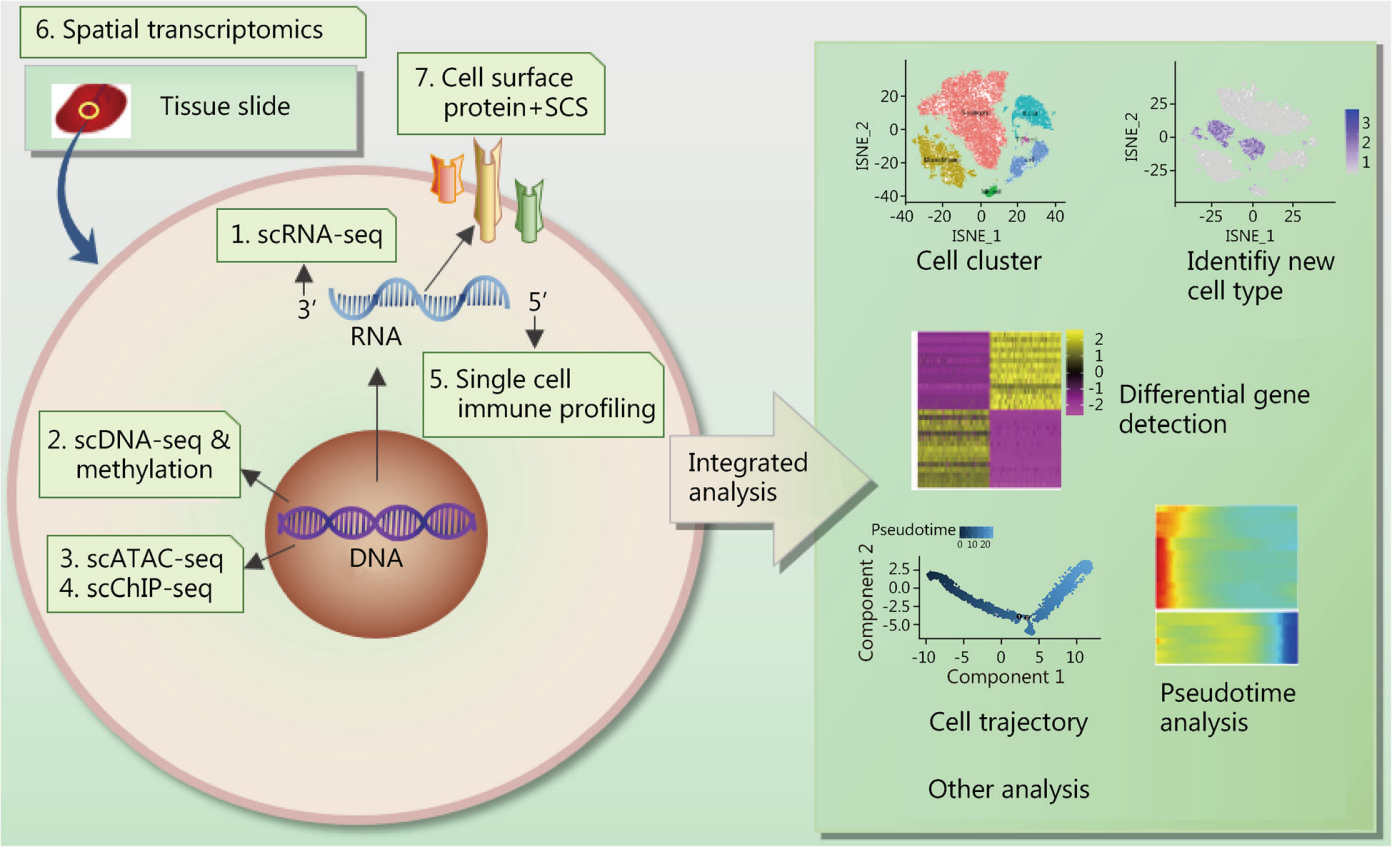
Overview of current common SCS approaches and analyses. 1. scRNA-seq***:*** By capturing transcripts and generating sequencing libraries for individual cells, scRNA-seq assesses biological properties of cell populations at unprecedented resolution. Several platforms are available, including Drop-seq, Smart-seq2, MARS-seq, and 10X Genomics. 2. scDNA-seq & methylation: Also known as single-cell genomic sequencing, whole genomic DNA from individual cells is captured, amplified, and sequenced. scDNA-seq can provide information on copy numbers and single nucleotide variants (SNVs). 3. scATAC-seq: A single-cell assay for transposase-accessible chromatin sequencing, provides a way to understand the regulatory landscape of the genome. 4. scCHIP-seq: Single-cell chromatin immunoprecipitation sequencing reveals the epigenetic heterogeneity in individual cells. 5. Single-cell immune profiling: A comprehensive approach to simultaneously examine the cellular context of the adaptive immune response and immune repertoires of T and B cells on a cell-by-cell basis. This approach can reveal insights into T and B cell variable (V), diversity (D), and joining (J) genes, known as V(D)J recombination, and immune cell profiling. 6. Spatial transcriptomics: An array containing capture probes is employed to bind RNA from tissue sections, and cDNA is then synthesized, followed by preparation of sequence libraries. The libraries are then sequenced, providing information on what genes are expressed, the changing quantities, and where the cells derive from. 7. Cell surface protein + SCS: This approach simultaneously measures both gene and cell surface protein expression in the same cell. As shown in the right panel of the figure, by applying bioinformatics tools and computational analysis, this SCS technology can reveal complex and rare cell populations, uncover regulatory relationships between genes, track the trajectories of distinct cell lineages in development, and many other applications

In the early stage of the medicine, symptoms were usually the only clues for directing treatment. With the development of molecular biotechnology, scientists have begun to understand the molecular information hidden behind the symptoms. The emergence of bulk sequencing has provided an unbiased way to detect gene signatures and has promoted the Human Genome Project (HGP), bringing us into the postgenomics era during the past several decades. Today, SCS technologies lead medical research towards greater innovation and significant discoveries. Combined with bioinformatics analysis, we are able to substantially expand our knowledge in understanding cell-cell crosstalk and networking, identifying new cell phenotypes and subtypes, determining cell trajectory, and so on (Fig. 1). These findings can provide us with new insights into the mechanisms of lung homeostasis and diseases and thus suggest new diagnostic biomarkers and therapeutic targets.

### Cell diversity in lung development

Development of the lung into its specialized structure and cell types is highly regulated by tissue-specific growth factors, cytokines, transcription factors, and interaction with the immune compartment [20, 21]. The immune system in the lung consists of leukocytes migrating from hematopoietic sites and lung stromal cells. Proper immune component development is essential for homeostasis, and dysregulation of immune function may lead to disease, such as tissue inflammation, fibrosis, or cancer [22]. Studies with scRNA-seq analysis have provided us with paradigm changing insights into the dynamic alterations in cell diversity during lung development. Cohen et al. [18] profiled the immune and nonimmune lung cells by scRNA-seq, showing that cell composition varies widely along major timepoints in lung development, from the 12.5-day embryonic lung to postnatal 7 days. In the early embryo timepoint (E12.5), over 50% of immune cells are macrophages, while monocytes are the dominant cell type in the canalicular stage of lung development (E16.5). Later, all major immune cell populations are present in late pregnancy. On the postnatal 7th day, the lymphoid cell compartment, B cells and T cells, make up of 32% of the CD45^+^ population. These findings show the dynamic changes in cell components during development, which provide guidance for further studies on cellular dynamics, differentiation and maturation of the lung.

The alveolar macrophage (AM) is a self-maintaining tissue-specific cell type in lung tissue. Previous studies have shown that AMs originate from fetal liver embryonic precursors [23]. This theory has been confirmed by the slingshot trajectory analysis of scRNA-seq data of mouse lung, which showed that macrophages in the late embryonic phase and postnatal time form a continuous transcriptional spectrum with E16.5 monocytes [24, 25]. However, these AMs are mature macrophages in alveolae. As characterized by highly expressed peroxisome proliferator activated receptor gamma (PPARγ), AM gene signatures, including fatty acid binding protein 4 (FABP4), lipoprotein lipase (LPL), C-type lectin domain family 7 member A (CLEC7A), and integrin alpha X (ITGαX), are only observed in postnatal mice. ScRNA-seq data from mice show that not all of the macrophages that appear during lung development turn into AMs [18]. In early pregnancy, macrophages express high levels of chemokine (C-X3-C motif) receptor 1 (CX3CR1) and complement genes, including complement component 1 q subcomponents alpha and beta (C1qα, C1qβ). However, this type of macrophage is diminished in the later canalicular stage without differentiating into a mature AM. It is postulated that this type of macrophage might be involved in the mechanisms of some spontaneous pulmonary illnesses, although the actual effect of these macrophages is still unclear.

Transcriptomic advances at the single-cell level have revealed the presence of early, intermediate, and late myeloid cell precursors and their dynamic process of differentiation and maturation [21, 26, 27], while the transcriptional regulation of lung immune cells during development is still lacking. Regulatory network analysis in future studies would contribute to a better understanding of the physiological processes in the lung.

### scRNA-seq reveals specific phenotypes of myeloid cells in the lung

NGS approaches have largely enhanced our understanding of lung cell biology. Emerging data, mostly derived from scRNA-seq, support that resident tissue macrophages (RTMs) are a fully recognized heterogeneous population of immune cells exhibiting tissue-specific phenotypes and functions [28]. In the murine lung, AMs are the major population in alveolar spaces, while a minor population of interstitial macrophages (IMs) reside within the lung parenchyma. Transcriptome analysis identified that AMs highly express keratin 79 (KRT79), keratin 19 (KRT19), and carbonic anhydrase 4 (CAR4) compared with expression in other types of macrophages [29]. Human AMs, however, exhibit poor antigen presentation function due to the lack of expression of costimulatory molecules, such as CD86 [30]. In addition, murine AMs show decreased phagocytic activity compared with that of lung IMs [31] and express low levels of CD11b, which is related to integrin activity [32]. These data enhanced our understanding of the phenotypical and functional diversity in AMs.

Using scRNA-seq analysis, Chakarov et al. [33] identified two independent populations of IMs in mouse lungs that exhibited distinct gene expression profiles, Lyve1^lo^MHCII^hi^CX3CR1^hi^ IM and Lyve1^hi^MHCII^lo^CX3CR1^lo^ IM. Both of these IM populations are involved in lung immune responses. Lyve1^lo^MHCII^hi^CX3CR1^hi^ IMs are mostly found surrounding nerves and have a higher antigen presentation function, whereas Lyve1^hi^MHCII^lo^CX3CR1^lo^ IMs are often closely associated with blood vessels across tissues, affecting wound and tissue repair. Evidence shows that, similar to the RTMs, these two subtypes of IM are from two separate lineages arising from tissue-recruited monocytes [33, 34].

Massively parallel scRNA-seq (MARS-seq) analysis of basophils from mouse lung and peripheral blood showed that lung basophils, compared with blood basophils, present a unique gene signature, including the expression of interleukin 6 (IL-6), interleukin 13 (IL-13), chemokine (C-X-C motif) ligand 2 (CXCL2), tumor necrosis factor (TNF) and chemokine (C-C motif) ligand 4 (CCL4) [18]. These phenotypic changes in specific gene signatures in lung basophils represent functional shifting. AMs from lung basophil-depleted mice shared some genes with immature macrophages and showed deficiency in anti-inflammatory ability and phagocytic properties, suggesting that the changes in the basophil gene signature are important to the differentiation and phagocytic properties of macrophages through basophil-macrophage interaction [18, 35].

Canonical neutrophil transcriptional markers include matrix metallopeptidase 8 (MMP-8), matrix metallopeptidase 9 (MMP-9), S100 calcium binding protein A8 (S100A8), and S100 calcium binding protein A9 (S100A9) [21, 36, 37]. scRNA-seq revealed that pulmonary neutrophils display high expression of the Retnlg gene [18], encoding resistin-like gamma, which has been found in the nasal respiratory epithelium [38] and bone marrow [39]. Interestingly, Retnlg is detected at low levels in granulocytes in peripheral blood [39]. Studies on the function of Retnlg are lacking. Since Retnlg is usually found in the extracellular region or secreted in plasma [40], it is speculated that it may potentially promote chemotaxis of myeloid cells [41]. Although the function of Retnlg in neutrophils is still unclear, high expression of Retnlg in pulmonary neutrophils may suggest a role for these neutrophils in further inducing myeloid cells into the lung, which may contribute to amplified innate immune cell infiltration in the lung in response to inflammation.

Emerging data showed a novel neutrophil population in the lung that is able to reverse migrate from the inflamed loci to circulation termed reverse migration neutrophils [42, 43]. Reverse migration neutrophils with the phenotype CXCR1^low^ICAM1^high^ [44] differ from CXCR1^high^ICAM1^low^ neutrophils in the blood and CXCR1^low^ICAM1^high^ neutrophils in tissue [45]. The mechanism underlying neutrophil reverse migration from the lung remains unclear. It is speculated that the lung modulates neutrophil phenotype to promote neutrophil clearance, including the mechanism of neutrophil homing to bone marrow via reverse migration [43].

### Myeloid cell transcriptional alterations in acute lung injury

Acute lung injury (ALI) and its more severe form acute respiratory distress syndrome (ARDS) are heterogeneous syndromes with diverse sets of etiologies and outcomes. Distinct alterations in macrophages and neutrophils in ALI have been reported in recent decades. The different roles of AM subtypes, M1 and M2, in the development and resolution of ALI have been well accepted since 1986 [46, 47]. In general, M1 AMs play an important role in promoting acute lung inflammation and subsequent lung injury by releasing various proinflammatory mediators and inducing expression of factors mediating neutrophil recruitment into the lungs. In contrast, M2 AMs are considered an anti-inflammatory force involved in diminishing lung inflammation and resolution of ALI [48].

Next-generation RNA sequencing provides an unbiased way to reveal new regulatory mechanisms of lung inflammation by showing dynamic changes in the transcriptome. Halstead et al. [49] established an influenza A virus infection mouse model and explored transcript alterations in AMs by RNA sequencing. The results show that granulocyte-macrophage colony-stimulating factor (GM-CSF) derived from epithelial cells redirects AMs from an “M1-like” state to a more “M2-like” activation state. Secretion of GM-CSF from epithelial cells is decreased in response to cell damage by pathogen-associated molecule patterns (PAMPs) or damage-associated molecule patterns (DAMPs), and the decrease in GM-CSF secretion results in a decrease in M2 AMs.

Delayed neutrophil apoptosis has been regarded as one of the mechanisms that induces a persistent inflammatory response [50]. However, RNA sequencing from the mouse *Yersinia pestis* infection model shows that in inflammatory lesions of pneumonic plague, neutrophil survival depends on not only the apoptosis mechanism but also the type III secretion system effector YopM [51].

Data from NGS not only confirm the upregulation of cytokine- and chemokine-related genes in response to lipopolysaccharides (LPS) [52], but also reveal alterations in genes related to other pathological signaling pathways. In the ventilator-induced lung injury animal model, RNA sequencing of lung tissue showed that activation of the mechanistic target of rapamycin pathway and Janus kinase-signal transducer and activator of transcription (JAK/STAT) signaling were implicated in early inflammation, while the hypoxia inducible factor-1 (HIF-1) and nuclear factor κB (NF-κB) signaling pathways were activated in the late stage, which might be related to subsequent fibrosis [53]. Owing to the unbiased sequencing method, some undefined genes have been found to be related to ALI occurrence. Kangelaris et al. [54] compared whole blood RNA from sepsis patients with or without ARDS. The results showed that haptoglobin (HP) and resistin (RETN) were significantly upregulated in the ARDS patients, while hydroxycarboxylic acid receptor 3 (HCAR3), retinol binding protein 7 (RBP7), and membrane metallo-endopeptidase (MME) were decreased. These findings provide new study targets to illuminate the occurrence of ALI in future research.

Since NGS detects the sequences in an unbiased way, NGS also enhances our knowledge about noncoding RNAs, such as long noncoding RNA (lncRNA), microRNA (miRNA) and circular RNA (circRNA), which were considered useless products of RNA splicing errors [55]. Recently, noncoding RNAs were found to be involved in the regulation of many vital physiological and pathological processes [56]. Using NGS, Ye et al. [57] found ten circRNAs that were differentially expressed between rats with ALI induced by smoke inhalation and the control group, providing an important basis for research and future studies of circRNAs in ALI.

By employing NGS technology, it has been found that the gene expression and the enriched pathways were significantly changed in ALI. However, the results significantly differed, and there is still no conclusion about what main factors play crucial roles in the process of ALI. This might be due to the heterogeneity of risk factors and etiologies, in addition to cell heterogeneity. With the development of scRNA-seq technology, further studies will reveal the determinant factors and the cell networking mechanisms underlying the progression of ALI.

### Myeloid cell transcriptional alterations in chronic lung disease

Lung fibrosis is often a late stage process in many lung diseases, such as those caused by toxicity and infection. In a prevailing theory, lung fibrosis occurs due to fibroblast proliferation after an initial insult to the alveolar epithelium and subsequent proinflammatory response [58]. Fibroblasts invade the epithelial layer to plug wounds. However, in some cases, fibroblasts fail to undergo apoptosis and continue to generate stiff tissue, resulting in irreversible alveolar collapse. The mechanisms of lung fibrosis are not fully elucidated but are believed to be a result of the interaction of many factors. Using NGS approaches, genetic studies on pulmonary fibrosis have made great progress, most of which have led to the discovery of mutations in genes related to telomere homeostasis [59]. Whole-lung transcriptome profiling showed dysregulated canonical pathways in the fibrosis mouse model, including the pathways for bacteria/virus recognition, inflammation, leukocyte extravasation, and ROS production [60]. By using scRNA-seq, Peyser et al. [61] found that early events in lung fibrosis might not involve significant changes in fibroblast number, while the numbers of macrophages, dendritic cells (DCs), and proliferating myeloid cells are increased. These increased cells may be involved in fibrosis pathogenesis. Distinct monocyte and macrophage subtypes have been found in the development of fibrosis [62, 63]. Reyfman et al. [64] performed scRNA-seq on lung tissue obtained from eight transplant donors and their eight counterpart recipients with pulmonary fibrosis. The data revealed that AM genes originating from the lungs of patients with fibrosis were enriched in “exocytosis”, “secretion”, “regulation of cell migration” and “extracellular matrix organization”. By using scRNA-seq on a bleomycin-induced lung fibrosis mouse model, Aran et al. [63] identified a profibrotic macrophage subpopulation expressing the specific markers CX3CR1 and SiglecF, which localized at the sites of fibrotic scarring where Pdgfra^+^ and Pdgfrb^+^ fibroblasts accumulated. This finding suggests an important role of the macrophage subpopulation in the regulation of fibroblasts. Regarding the source of the profibrotic macrophages, the study showed that the macrophages partially shared a gene expression profile with both alveolar and interstitial macrophages, suggesting a transitional state of resident lung macrophages that is initiated following injury. Not only macrophages but also distinct monocytes, characterized by Ceacam1^+^Msr1^+^Ly6C^−^F4/80^−^Mac1^+^ and termed segregated-nucleus-containing atypical monocytes (SatMs), were also found in the bleomycin-induced fibrosis mouse model, suggesting a role for these cells in the progression of fibrosis. Notably, the differentiation of SatMs was dependent on CCAAT/enhancer binding protein β (C/EBPβ), which usually plays a crucial role in the maturation and differentiation of granulocytes [37]. These results indicate that targeting myeloid cells is a potential novel strategy for the prevention and therapy of lung fibrosis.

COPD is a common outcome of chronic lung inflammation, characterized by reduced lung function. With regard to the infiltrating myeloid cells, there are two main phenotypes of COPD, neutrophil-associated COPD and eosinophil-associated COPD [65]. Neutrophilic inflammation is the most common inflammatory phenotype in COPD, which is mainly activated by proinflammatory mediators and DAMPs released by epithelial cells and resident macrophages under stimuli [66]. Recruited neutrophils subsequently release proteases, induce airway damage, and activate adaptive immune Th1 and Th17 cells [66, 67]. For eosinophil-associated COPD, patients usually show a higher risk of severe exacerbations [68]. NGS data have now shown a greater diversity of COPD. Genome-wide association studies (GWAS) on COPD patients have identified several COPD-associated genome variants, suggesting that COPD is a disease with genetic predisposition and altered immunity [69, 70]. By analyzing large-airway epithelium, alveolar macrophages, and peripheral blood samples from COPD patients by RNA-seq, Morrow et al. [71] observed a significant overlap in genes from large-airway epithelium and macrophage for smoking and airway disease phenotypes, including cytochrome P450 family 1 subfamily B member 1 (CYP1B1) and aryl-hydrocarbon receptor repressor (AHRR). CYP1B1 is involved in oxidative metabolism, while AHRR mediates dioxin toxicity due to its DNA binding effect. These results highlight the shared tissue-specific signatures of lung disease and damage. Furthermore, RNA-seq profiling revealed some significantly associated emphysema genes, including asparaginase-like 1 (ASRGL1), latrophilin 2 (LPHN2), and endothelin receptor type B (EDNRB) [72]. Of note, most studies employed nasal and bronchial brushing samples from COPD patients, and thus, the samples were a mix of multi-cell populations, which should include epithelial cells, alveolar macrophages, infiltrated neutrophils and many others. Studies using mixed-cell samples may reveal genes for COPD susceptibility and identify biomarkers for diagnosis and prediction; however, these studies fail to elucidate the roles of different cells in the process of COPD.

### Myeloid cell heterogeneity in lung cancer

Tumor heterogeneity is a significant cause of the poor therapeutic effects of chemotherapy and radiotherapy. SCS technology provides a better approach to studying tumor microenvironments and heterogeneity. Immune cells show distinct gene signatures in tumor patients in high or low risk groups in terms of overall survival [73]. In addition, intratumoral immune cell densities, including dentric cells (DCs), neutrophils, macrophages and CD8^+^ T cells, were associated with molecular alterations in lung adenocarcinoma patients, underlying the interactions between tumor cells and their microenvironment [74]. The heterogeneity of both tumor cells and immune cells have potential impacts on the efficacy of immunotherapy, especially for optimal personalized immunotherapy [75–77]. Studies show that peripheral blood immune cells are altered in lung cancer patients [78]. Zilionis et al. [19] compared tumor-infiltrating myeloid cells and peripheral blood immune cells of the same patients by scRNA-seq, and the data showed only partial overlap in the states of the two groups, suggesting that immune populations can be reprogrammed by the tumor microenvironment [79]. Considering that tumor microenvironments are varied, several studies have analyzed the tumor-infiltrating populations in nonsmall cell lung cancer (NSCLC) by scRNA-seq. The studies revealed distinct phenotypes of neutrophils, monocytes, and macrophages in patients and mouse models [19, 76, 80, 81].

Neutrophils have been regarded as a kind of terminally differentiated cell and thus have been assumed to have less heterogeneity. However, scRNA-seq revealed the transcriptional plasticity of neutrophils, which express significant phenotypes in NSCLC [82]. These phenotypes play both protumoral and antitumoral roles [83, 84]. The distinct phenotypes could be the result of the development of specific progenitor cells or reprogramming of the tumor microenvironment. In bone marrow, Zhu et al. [36] found that Lin^−^CD117^+^Ly6a/e^−^ cells were a distinct neutrophil progenitor population with protumoral activity. In the lung, neutrophils that highly express sialic acid binding Ig-like lectin F (SiglecF) have been found in mice with tumors and exhibit several protumor functions [85], while SiglecF^low^ cells are found in tumor-free lungs. SiglecF^high^ neutrophils overexpressed colony stimulating factor 1 (CSF1), leukotriene C4 synthase (LTC4S), runt related transcription factor 1 (RUNX1), secreted phosphoprotein 1 (SPP1), vascular endothelial growth factor A (VEGFA), and X-box binding protein 1 (XBP1) transcripts [19]. These molecules have been previously found in tumor cells and have been identified as playing protumoral roles [86–88]. A new study showed that CCL3, cystatin B (CSTB), cathepsin B (CTSB), and interleukin-1 receptor-associated kinase 2 (IRAK2), molecules that are involved in the inflammatory response of neutrophils, are specifically found in neutrophils from mice with tumors [19]. To our knowledge, these transcripts may not be able to promote cell proliferation and/or differentiation. However, they are potentially involved in the development of the immune microenvironment and provide an increased probability for tumor immune escape.

Traditionally, two types of DCs are found in mice. Ly6c^high^Ccr2^+^CX3CR1^int^ monocytes, which can extravasate into tissues and give rise to macrophages and DCs, and Ly6c^low^Ccr2^−^CX3CR1^high^ monocytes, which remain in the vasculature [79]. These two types correspond to the classical subsets in humans, CD14^+^ monocytes and CD14^−^CD16^+^ monocytes. In addition, a study using scRNA-seq reported that a subtype of “neutrophil-like” monocyte that expresses a set of neutrophil-associated genes was found in human blood [89]. A recent report further showed that monocytes expressing S100A8, S100A9, and colony stimulating factor 3 receptor (CSF3R) were found in both human and mouse lung cancer tissue [19]. Whether the “neutrophil-like” monocytes are protumoral or antitumoral remains unclear.

Tumor-infiltrating macrophages from human lung cancer biopsy showed distinct expression of chemokines, including the neutrophil chemoattractant CXCL5 and the T cell recruiting chemokine CXCL9 [19]. These chemokines may serve as migration cues for other immune cells, resulting in cell population diversity in the tumor microenvironment. Moreover, monocyte-to-M2 differentiation was found to be a prevalent trajectory in tumor progression [80].

In summary, studies with scRNA-seq have identified new phenotypes and gene signatures related to tumor processes and have explored the diversity of myeloid cells in the tumor microenvironment. Tumor therapies that target immune cells have shown clinical benefits, indicating that immune cells are key regulators of cancer growth [90]. Further studies in this area will provide us with new means of diagnosis and treatment.

## Conclusions

The pulmonary immune environment is an intricate network composed of various interacting cell types. NGS technology has revealed myeloid cell heterogeneity and identified specific cell phenotypes in the lung during development, homeostasis, and diseases. NGS has also explored biomarkers for the diagnosis and prediction of lung disease prognosis. However, identification of the main regulatory factors and reprogramming mechanisms of immune cells in lung development and diseases is still limited. With the advance of high-throughput sequencing, future studies will be able to map a more detailed portrait of gene expression and regulatory networks in the lung.

SCS techniques, represented by single-cell DNA sequencing, scRNA-seq, and single-cell epigenomic sequencing, provide us with a broad range of cellular parameters, including DNA and RNA sequences, DNA methylation, and chromatin accessibility. Today, SCS technology is being quickly developed. For example, recent efforts have pioneered methods to record spatial information. The majority of current studies are focused on measurements of a single modality. The combinatorially barcoded profiling technology makes it possible to combine multimodal technologies. With the help of integrative computational methods, it will be possible to build a comprehensive molecular view of cells and tissue states based on the substantial information derived from SCS. NGS will, no doubt, be largely beneficial in finding new targets for the diagnosis and treatment of lung diseases.

## Data Availability

Not applicable.

## Abbreviations

AHRR: Aryl-hydrocarbon receptor repressor
ALI: Acute lung injury
AM: Alveolar macrophage
ARDS: Acute respiratory distress syndrome
ASRGL1: Asparaginase-like 1
C/EBPβ: CCAAT/enhancer binding protein β
C1qα: Complement component 1 q subcomponent alpha
C1qβ: Complement component 1 q subcomponent beta
CAR4: Carbonic anhydrase 4
CCL4: Chemokine (C-C motif) ligand 4
CLEC7A: C-type lectin domain family 7 member A
COPD: Chronic obstructive pulmonary disease
CSF1: Colony stimulating factor 1
CSF3R: Colony stimulating factor 3 receptor
CSTB: Cystatin B
CTC: Circulating tumor cell
CTSB: Cathepsin B
CX3CR1: Chemokine (C-X3-C motif) receptor 1
CXCL2: Chemokine (C-X-C motif) ligand 2
CYP1B1: Cytochrome P450 family 1 subfamily B member 1
DAMPs: Damage-associated molecule patterns
DCs: Dendritic cells
EDNRB: Endothelin receptor type B
FABP4: Fatty acid binding protein 4
GM-CSF: Granulocyte-macrophage colony-stimulating factor
GWAS: Genome-wide association studies
HCAR3: Hydroxycarboxylic acid receptor 3
HGP: Human Genome Project
HIF-1: Hypoxia inducible factor-1
HP: Haptoglobin
Icam1: Intercellular adhesion molecule 1
IL-13: Interleukin 13
IL-6: Interleukin 6
IMs: Interstitial macrophages
IRAK2: Interleukin-1 receptor-associated kinase 2
ITGαX: Integrin alpha X
JAK/STAT: Janus kinase-signal transducer and activator of transcription
KEGG: Kyoto Encyclopedia of Genes and Genomes
KRT19: Keratin 19
KRT79: Keratin 79
LPHN2: Latrophilin 2
LPL: Lipoprotein lipase
LPS: Lipopolysaccharides
LTC4S: Leukotriene C4 synthase
Lyve1: Lymphatic vessel endothelial hyaluronan receptor 1
MARS-seq: Massively parallel scRNA-seq
MHC II: Major histocompatibility complex class II
MME: Membrane metallo-endopeptidase
MMP-8: Matrix metallopeptidase 8
MMP-9: Matrix metallopeptidase 9
NF-κB: Nuclear factor κB
NGS: Next generation sequencing
NSCLC: Nonsmall cell lung cancer
PAMPs: Pathogen-associated molecule patterns
PPARγ: Peroxisome proliferator activated receptor gamma
RBP7: Retinol binding protein 7
RETN: Resistin
ROS: Reactive oxygen species
RTMs: Resident tissue macrophages
RUNX1: Runt related transcription factor 1
S100A8: S100 calcium binding protein A8
S100A9: S100 calcium binding protein A9
SatMs: Segregated-nucleus-containing atypical monocytes
ScRNA-seq: Single-cell RNA sequencing
SCS: Single-cell sequencing
SiglecF: Sialic acid binding Ig-like lectin F
SPP1: Secreted phosphoprotein 1
TNF: Tumor necrosis factor
VEGFA: Vascular endothelial growth factor A
XBP1: X-box binding protein 1

## References

[1] Man WH, de Steenhuijsen Piters WAA, Bogaert D (2017). The microbiota of the respiratory tract: gatekeeper to respiratory health. Nat Rev Microbiol.

[2] Aulakh GK (2018). Neutrophils in the lung: “the first responders”. Cell Tissue Res.

[3] Brown M, Assen FP, Leithner A, Abe J, Schachner H, Asfour G (2018). Lymph node blood vessels provide exit routes for metastatic tumor cell dissemination in mice. Science..

[4] Yipp BG, Kim JH, Lima R, Zbytnuik LD, Petri B, Swanlund N, et al. The lung is a host defense niche for immediate neutrophil-mediated vascular protection. Sci Immunol. 2017;2. pii: eaam8929. 10.1126/sciimmunol.aam8929 PMC5472445 28626833

[5] Riches DWH, Martin TR (1809). Overview of innate lung immunity and inflammation. Methods Mol Biol.

[6] Amulic B, Cazalet C, Hayes GL, Metzler KD, Zychlinsky A (2012). Neutrophil function: from mechanisms to disease. Annu Rev Immunol.

[7] Ng LG, Ostuni R, Hidalgo A (2019). Heterogeneity of neutrophils. Nat Rev Immunol..

[8] Sanger F, Air GM, Barrell BG, Brown NL, Coulson AR, Fiddes CA (1977). Nucleotide sequence of bacteriophage phi X174 DNA. Nature..

[9] Sanger F, Nicklen S, Coulson AR (1977). DNA sequencing with chain-terminating inhibitors. Proc Natl Acad Sci U S A.

[10] McPherson JD (2009). Next-generation gap. Nat Methods.

[11] Schuster SC (2008). Next-generation sequencing transforms today’s biology. Nat Methods.

[12] Anderson MW, Schrijver I (2010). Next generation DNA sequencing and the future of genomic medicine. Genes (Basel).

[13] Zhao M, Liu D, Qu H (2017). Systematic review of next-generation sequencing simulators: computational tools, features and perspectives. Brief Funct Genomics.

[14] Behjati S, Tarpey PS (2013). What is next generation sequencing?. Arch Dis Child Educ Pract Ed.

[15] Wang Y, Navin NE (2015). Advances and applications of single-cell sequencing technologies. Mol Cell.

[16] Stuart T, Satija R (2019). Integrative single-cell analysis. Nat Rev Genet.

[17] Schultze JL, Mass E, Schlitzer A (2019). Emerging principles in myelopoiesis at homeostasis and during infection and inflammation. Immunity..

[18] Cohen M, Giladi A, Gorki A-D, Solodkin DG, Zada M, Hladik A (2018). Lung single-cell signaling interaction map reveals basophil role in macrophage imprinting. Cell.

[19] Zilionis R, Engblom C, Pfirschke C, Savova V, Zemmour D, Saatcioglu HD (2019). Single-cell transcriptomics of human and mouse lung cancers reveals conserved myeloid populations across individuals and species. Immunity.

[20] Panduro M, Benoist C, Mathis D (2016). Tissue Tregs. Annu Rev Immunol.

[21] Paul F, Arkin Y, Giladi A, Jaitin DA, Kenigsberg E, Keren-Shaul H (2015). Transcriptional heterogeneity and lineage commitment in myeloid progenitors. Cell..

[22] Farber DL, Sims PA (2019). Dissecting lung development and fibrosis at single-cell resolution. Genome Med.

[23] Hashimoto D, Chow A, Noizat C, Teo P, Beasley MB, Leboeuf M (2013). Tissue-resident macrophages self-maintain locally throughout adult life with minimal contribution from circulating monocytes. Immunity..

[24] Ginhoux F (2014). Fate PPAR-titioning: PPAR-γ “instructs” alveolar macrophage development. Nat Immunol.

[25] Guilliams M, De Kleer I, Henri S, Post S, Vanhoutte L, De Prijck S (2013). Alveolar macrophages develop from fetal monocytes that differentiate into long-lived cells in the first week of life via GM-CSF. J Exp Med.

[26] Velten L, Haas SF, Raffel S, Blaszkiewicz S, Islam S, Hennig BP (2017). Human haematopoietic stem cell lineage commitment is a continuous process. Nat Cell Biol.

[27] Olsson A, Venkatasubramanian M, Chaudhri VK, Aronow BJ, Salomonis N, Singh H (2016). Single-cell analysis of mixed-lineage states leading to a binary cell fate choice. Nature..

[28] Ginhoux F, Guilliams M (2016). Tissue-resident macrophage ontogeny and homeostasis. Immunity..

[29] Gautier EL, Shay T, Miller J, Greter M, Jakubzick C, Ivanov S (2012). Gene-expression profiles and transcriptional regulatory pathways that underlie the identity and diversity of mouse tissue macrophages. Nat Immunol.

[30] Blumenthal RL, Campbell DE, Hwang P, DeKruyff RH, Frankel LR, Umetsu DT (2001). Human alveolar macrophages induce functional inactivation in antigen-specific CD4 T cells. J Allergy Clin Immunol.

[31] Hussell T, Bell TJ (2014). Alveolar macrophages: plasticity in a tissue-specific context. Nat Rev Immunol..

[32] Zaynagetdinov R, Sherrill TP, Kendall PL, Segal BH, Weller KP, Tighe RM (2013). Identification of myeloid cell subsets in murine lungs using flow cytometry. Am J Respir Cell Mol Biol.

[33] Chakarov S, Lim HY, Tan L, Lim SY, See P, Lum J, et al. Two distinct interstitial macrophage populations coexist across tissues in specific subtissular niches. Science. 2019;363. pii: eaau0964. 10.1126/science.aau0964 30872492

[34] Lavin Y, Winter D, Blecher-Gonen R, David E, Keren-Shaul H, Merad M (2014). Tissue-resident macrophage enhancer landscapes are shaped by the local microenvironment. Cell..

[35] Shibata S, Miyake K, Tateishi T, Yoshikawa S, Yamanishi Y, Miyazaki Y (2018). Basophils trigger emphysema development in a murine model of COPD through IL-4-mediated generation of MMP-12-producing macrophages. Proc Natl Acad Sci U S A.

[36] Zhu YP, Padgett L, Dinh HQ, Marcovecchio P, Blatchley A, Wu R (2018). Identification of an early unipotent neutrophil progenitor with pro-tumoral activity in mouse and human bone marrow. Cell Rep.

[37] Giladi A, Paul F, Herzog Y, Lubling Y, Weiner A, Yofe I (2018). Single-cell characterization of haematopoietic progenitors and their trajectories in homeostasis and perturbed haematopoiesis. Nat Cell Biol.

[38] Gerstmayer B, Küsters D, Gebel S, Müller T, Van Miert E, Hofmann K (2003). Identification of RELMγ, a novel resistin-like molecule with a distinct expression pattern. Genomics..

[39] Schinke T, Haberland M, Jamshidi A, Nollau P, Rueger JM, Amling M (2004). Cloning and functional characterization of resistin-like molecule γ. Biochem Biophys Res Commun.

[40] Shojima N, Ogihara T, Inukai K, Fujishiro M, Sakoda H, Kushiyama A (2005). Serum concentrations of resistin-like molecules beta and gamma are elevated in high-fat-fed and obese db/db mice, with increased production in the intestinal tract and bone marrow. Diabetologia..

[41] Chumakov AM, Kubota T, Walter S, Koeffler HP (2004). Identification of murine and human XCP1 genes as C/EBP-epsilon-dependent members of FIZZ/Resistin gene family. Oncogene..

[42] Colom B, Bodkin JV, Beyrau M, Woodfin A, Ody C, Rourke C (2015). Leukotriene B4-neutrophil elastase axis drives neutrophil reverse transendothelial cell migration *in vivo*. Immunity..

[43] Wang J, Hossain M, Thanabalasuriar A, Gunzer M, Meininger C, Kubes P (2017). Visualizing the function and fate of neutrophils in sterile injury and repair. Science..

[44] Buckley CD, Ross EA, McGettrick HM, Osborne CE, Haworth O, Schmutz C (2006). Identification of a phenotypically and functionally distinct population of long-lived neutrophils in a model of reverse endothelial migration. J Leukoc Biol.

[45] de Oliveira S, Rosowski EE, Huttenlocher A (2016). Neutrophil migration in infection and wound repair: going forward in reverse. Nat Rev Immunol.

[46] Gordon S (2003). Alternative activation of macrophages. Nat Rev Immunol..

[47] Bitterman PB, Wewers MD, Rennard SI, Adelberg S, Crystal RG (1986). Modulation of alveolar macrophage-driven fibroblast proliferation by alternative macrophage mediators. J Clin Invest.

[48] Johnston LK, Rims CR, Gill SE, McGuire JK, Manicone AM (2012). Pulmonary macrophage subpopulations in the induction and resolution of acute lung injury. Am J Respir Cell Mol Biol.

[49] Halstead ES, Umstead TM, Davies ML, Kawasawa YI, Silveyra P, Howyrlak J (2018). GM-CSF overexpression after influenza a virus infection prevents mortality and moderates M1-like airway monocyte/macrophage polarization. Respir Res.

[50] Fan J (2010). TLR cross-talk mechanism of hemorrhagic shock-primed pulmonary neutrophil infiltration. Open Crit Care Med J.

[51] Stasulli NM, Eichelberger KR, Price PA, Pechous RD, Montgomery SA, Parker JS (2015). Spatially distinct neutrophil responses within the inflammatory lesions of pneumonic plague. MBio..

[52] Wan QQ, Wu D, Ye QF (2018). Candidate genes as biomarkers in lipopolysaccharide-induced acute respiratory distress syndrome based on mRNA expression profile by next-generation RNA-seq analysis. Biomed Res Int.

[53] Wang L, Zhang N, Zhang Y, Xia J, Zhan Q, Wang C (2018). Landscape of transcription and long non-coding RNAs reveals new insights into the inflammatory and fibrotic response following ventilator-induced lung injury. Respir Res.

[54] Kangelaris KN, Prakash A, Liu KD, Aouizerat B, Woodruff PG, Erle DJ (2015). Increased expression of neutrophil-related genes in patients with early sepsis-induced ARDS. Am J Physiol Lung Cell Mol Physiol.

[55] Sanger HL, Klotz G, Riesner D, Gross HJ, Kleinschmidt AK (1976). Viroids are single-stranded covalently closed circular RNA molecules existing as highly base-paired rod-like structures. Proc Natl Acad Sci U S A.

[56] Wan L, Zhang L, Fan K, Cheng ZX, Sun QC, Wang JJ (2016). Circular RNA-ITCH suppresses lung cancer proliferation *via* inhibiting the Wnt/β-catenin pathway. Biomed Res Int.

[57] Ye Z, Liu X, Yang Y, Zhang X, Yu T, Li S (2018). The differential expression of novel circular RNAs in an acute lung injury rat model caused by smoke inhalation. J Physiol Biochem.

[58] King TE, Pardo A, Selman M (2011). Idiopathic pulmonary fibrosis. Lancet..

[59] Borie Raphael, Kannengiesser Caroline, Sicre de Fontbrune Flore, Gouya Laurent, Nathan Nadia, Crestani Bruno (2017). Management of suspected monogenic lung fibrosis in a specialised centre. European Respiratory Review.

[60] Garcia AM, Allawzi A, Tatman P, Hernandez-Lagunas L, Swain K, Mouradian G (2018). R213G polymorphism in SOD3 protects against bleomycin-induced inflammation and attenuates induction of proinflammatory pathways. Physiol Genomics.

[61] Peyser R, MacDonnell S, Gao Y, Cheng L, Kim Y, Kaplan T (2019). Defining the activated fibroblast population in lung fibrosis using single-cell sequencing. Am J Respir Cell Mol Biol.

[62] Satoh T, Nakagawa K, Sugihara F, Kuwahara R, Ashihara M, Yamane F (2017). Identification of an atypical monocyte and committed progenitor involved in fibrosis. Nature..

[63] Aran D, Looney AP, Liu L, Wu E, Fong V, Hsu A (2019). Reference-based analysis of lung single-cell sequencing reveals a transitional profibrotic macrophage. Nat Immunol.

[64] Reyfman PA, Walter JM, Joshi N, Anekalla KR, McQuattie-Pimentel AC, Chiu S (2018). Single-cell transcriptomic analysis of human lung provides insights into the pathobiology of pulmonary fibrosis. Am J Respir Crit Care Med.

[65] Brightling Christopher, Greening Neil (2019). Airway inflammation in COPD: progress to precision medicine. European Respiratory Journal.

[66] Caramori G, Casolari P, Barczyk A, Durham AL, Di Stefano A, Adcock I (2016). COPD immunopathology. Semin Immunopathol.

[67] Doe C, Bafadhel M, Siddiqui S, Desai D, Mistry V, Rugman P (2010). Expression of the T helper 17-associated cytokines IL-17A and IL-17F in asthma and COPD. Chest..

[68] Bafadhel M, Peterson S, De Blas MA, Calverley PM, Rennard SI, Richter K (2018). Predictors of exacerbation risk and response to budesonide in patients with chronic obstructive pulmonary disease: a post-hoc analysis of three randomised trials. Lancet Respir Med.

[69] Sakornsakolpat P, Prokopenko D, Lamontagne M, Reeve NF, Guyatt AL, Jackson VE (2019). Genetic landscape of chronic obstructive pulmonary disease identifies heterogeneous cell-type and phenotype associations. Nat Genet.

[70] Wain LV, Shrine N, Artigas MS, Erzurumluoglu AM, Noyvert B, Bossini-Castillo L (2017). Genome-wide association analyses for lung function and chronic obstructive pulmonary disease identify new loci and potential druggable targets. Nat Genet.

[71] Morrow JD, Chase RP, Parker MM, Glass K, Seo M, Divo M (2019). RNA-sequencing across three matched tissues reveals shared and tissue-specific gene expression and pathway signatures of COPD. Respir Res.

[72] Obeidat M, Nie Y, Fishbane N, Li X, Bossé Y, Joubert P (2017). Integrative genomics of emphysema-associated genes reveals potential disease biomarkers. Am J Respir Cell Mol Biol.

[73] Li B, Cui Y, Diehn M, Li R (2017). Development and validation of an individualized immune prognostic signature in early-stage nonsquamous non-small cell lung cancer. JAMA Oncol.

[74] Mansuet-Lupo A, Alifano M, Pécuchet N, Biton J, Becht E, Goc J (2016). Intratumoral immune cell densities are associated with lung adenocarcinoma gene alterations. Am J Respir Crit Care Med.

[75] Kim KT, Lee HW, Lee HO, Kim SC, Seo YJ, Chung W (2015). Single-cell mRNA sequencing identifies subclonal heterogeneity in anti-cancer drug responses of lung adenocarcinoma cells. Genome Biol.

[76] Ma KY, Schonnesen AA, Brock A, Van Den Berg C, Eckhardt SG, Liu Z, et al. Single-cell RNA sequencing of lung adenocarcinoma reveals heterogeneity of immune response-related genes. JCI Insight. 2019;4. pii: 121387. 10.1172/jci.insight.121387 PMC6478414 30821712

[77] Karasaki T, Nagayama K, Kuwano H, Nitadori J, Sato M, Anraku M (2017). An immunogram for the cancer-immunity cycle: towards personalized immunotherapy of lung cancer. J Thorac Oncol.

[78] Lavin Y, Kobayashi S, Leader A, Amir E-AD, Elefant N, Bigenwald C (2017). Innate immune landscape in early lung adenocarcinoma by paired single-cell analyses. Cell.

[79] Ginhoux F, Jung S (2014). Monocytes and macrophages: developmental pathways and tissue homeostasis. Nat Rev Immunol..

[80] Song Q, Hawkins GA, Wudel L, Chou P-C, Forbes E, Pullikuth AK (2019). Dissecting intratumoral myeloid cell plasticity by single cell RNA-seq. Cancer Med.

[81] Damiani C, Maspero D, Di Filippo M, Colombo R, Pescini D, Graudenzi A (2019). Integration of single-cell RNA-seq data into population models to characterize cancer metabolism. PLoS Comput Biol.

[82] Stankovic B, Bjørhovde HAK, Skarshaug R, Aamodt H, Frafjord A, Müller E (2018). Immune cell composition in human non-small cell lung cancer. Front Immunol.

[83] Casbon AJ, Reynaud D, Park C, Khuc E, Gan DD, Schepers K (2015). Invasive breast cancer reprograms early myeloid differentiation in the bone marrow to generate immunosuppressive neutrophils. Proc Natl Acad Sci U S A.

[84] Hagerling C, Werb Z (2016). Neutrophils: critical components in experimental animal models of cancer. Semin Immunol.

[85] Engblom C, Pfirschke C, Zilionis R, Da Silva Martins J, Bos SA, Courties G, et al. Osteoblasts remotely supply lung tumors with cancer-promoting SiglecF^high^ neutrophils. Science. 2017;358. pii: eaal5081. 10.1126/science.aal5081 PMC6343476 29191879

[86] Hung JY, Horn D, Woodruff K, Prihoda T, LeSaux C, Peters J (2014). Colony-stimulating factor 1 potentiates lung cancer bone metastasis. Lab Investig.

[87] Kwon D, Koh J, Kim S, Go H, Min HS, Kim YA (2018). Overexpression of endoplasmic reticulum stress-related proteins, XBP1s and GRP78, predicts poor prognosis in pulmonary adenocarcinoma. Lung Cancer.

[88] Zhang Y, Du W, Chen Z, Xiang C (2017). Upregulation of PD-L1 by SPP1 mediates macrophage polarization and facilitates immune escape in lung adenocarcinoma. Exp Cell Res.

[89] Villani AC, Satija R, Reynolds G, Sarkizova S, Shekhar K, Fletcher J, et al. Single-cell RNA-seq reveals new types of human blood dendritic cells, monocytes, and progenitors. Science. 2017;356. pii: eaah4573. 10.1126/science.aah4573 PMC5775029 28428369

[90] Sharma P, Allison JP (2015). The future of immune checkpoint therapy. Science..

